# Genetic evaluation supports differential diagnosis in adolescent patients with delayed puberty

**DOI:** 10.1530/EJE-21-0387

**Published:** 2021-08-17

**Authors:** Tansit Saengkaew, Heena R Patel, Kausik Banerjee, Gary Butler, Mehul T Dattani, Michael McGuigan, Helen L Storr, Ruben H Willemsen, Leo Dunkel, Sasha R Howard

**Affiliations:** 1 Centre for Endocrinology, William Harvey Research Institute, Barts and the London School of Medicine and Dentistry, Queen Mary University of London, London, UK; 2 Endocrinology Unit, Department of Paediatrics, Faculty of Medicine, Prince of Songkla University, Songkhla, Thailand; 3 Department of Medicine and Health Sciences, Norwich Medical School, University of East Anglia, Norfolk, UK; 4 Department of Paediatrics, Barking, Havering and Redbridge University Hospitals NHS Trust, London, UK; 5 Department of Paediatric and Adolescent Endocrinology, University College London Hospital NHS Foundation Trust, London, UK; 6 UCL Great Ormond Street Institute of Child Health, University College London, London, UK; 7 Department of Paediatric Endocrinology, Great Ormond Street Hospital for Children NHS Foundation Trust, London, UK; 8 Department of Paediatrics, Countess of Chester NHS Foundation Trust, Chester, UK; 9 Department of Paediatric Endocrinology, Barts Health NHS Trust, London, UK

## Abstract

**Context:**

Pubertal delay can be the clinical presentation of both idiopathic hypogonadotropic hypogonadism (IHH) and self-limited delayed puberty (SLDP). Distinction between these conditions is a common but important diagnostic challenge in adolescents.

**Objective:**

To assess whether gene panel testing can assist with clinical differential diagnosis and to allow accurate and timely management of delayed puberty patients.

**Design:**

Retrospective study.

**Methods:**

Patients presenting with delayed puberty to UK Paediatric services, followed up to final diagnosis, were included. Whole-exome sequencing was analysed using a virtual panel of genes previously reported to cause either IHH or SLDP to identify rarely predicted deleterious variants. Deleterious variants were verified by *in silico* prediction tools. The correlation between clinical and genotype diagnosis was analysed.

**Results:**

Forty-six patients were included, 54% with a final clinical diagnosis of SLDP and 46% with IHH. Red flags signs of IHH were present in only three patients. Fifteen predicted deleterious variants in 12 genes were identified in 33% of the cohort, with most inherited in a heterozygous manner. A fair correlation between final clinical diagnosis and genotypic diagnosis was found. Panel testing was able to confirm a diagnosis of IHH in patients with pubertal delay. Genetic analysis identified three patients with IHH that had been previously diagnosed as SLDP.

**Conclusion:**

This study supports the use of targeted exome sequencing in the clinical setting to aid the differential diagnosis between IHH and SLDP in adolescents presenting with pubertal delay. Genetic evaluation thus facilitates earlier and more precise diagnosis, allowing clinicians to direct treatment appropriately.

## Introduction

Delayed puberty is a common problem in the paediatric endocrinology clinic, affecting over 2% of adolescents. This condition is diagnosed when children enter puberty 2–2.5 s.d. later than the population average (traditionally, after the age of 14 years in boys and 13 years in girls) (1). Several underlying aetiologies cause pubertal delay, including idiopathic hypogonadotropic hypogonadism (IHH) and hypergonadotropic hypogonadism. However, the most common cause of pubertal delay is self-limited, or constitutional, delayed puberty (SLDP), a functional hypogonadotropic state where individuals enter puberty late but are post-pubertal by the time they reach adulthood. Hypergonadotropic hypogonadism can be easily excluded by hormonal profiles. The differential diagnosis between SLDP and IHH, however, is often difficult, as both conditions may present with essentially the same clinical and hormonal features (2). Whilst a variety of clinical and biochemical investigations are available to make the diagnosis, none of these can reliably distinguish between those patients who will spontaneously enter and progress in a normal manner through puberty (i.e. SLDP), and those who will require medical induction of puberty and reproductive therapies (i.e. IHH) (3, 4).

This is a vital clinical distinction to make, as if IHH is diagnosed, treatment modalities to allow optimisation of future fertility (particularly for boys) can be used – in the form of gonadotropins rather than sex steroids for induction of puberty (5) and commenced earlier than the puberty induction regimen used for SLDP patients (6).

Observational studies have shown that SLDP is a familial condition, with the majority of pedigrees displaying autosomal dominant inheritance (with or without complete penetrance) (7, 8). Additionally, 79% of patients have a positive family history of SLDP without any family members with IHH, providing clinicians with further evidence of the diagnosis (7, 9, 10, 11). In contrast, IHH can be inherited via several modes of inheritance including autosomal dominant, autosomal recessive, X-linked or *de novo* mutation (12, 13). Moreover, in IHH families variable penetrance is commonly seen, probably due in part to the oligogenic inheritance of the disease (14, 15). To date, more than 40 genes have been identified that carry mutations which lead or contribute to conditions of IHH (13). Similarly, over the last 5 years, a smaller but increasing number of genes have been discovered that underlie SLDP by our and other groups through next-generation sequencing (16, 17). Crucially, whilst there is some overlap in the genetic background of these conditions, the majority of mutations are distinct between the two diseases (15). Therefore, genetic analysis using exome sequencing of a panel of known genes could be used to assist a clinician in distinguishing those adolescents with severe gonadotropin deficiency from those with isolated delayed puberty, allowing delivery of accurate and timely treatment to patients. However, this potential utility has yet to be assessed in a clinical cohort of patients presenting in adolescence with delayed puberty of undiagnosed aetiology.

Therefore, in this study, we investigated the burden of genetic variants in a real world, mixed ethnicity cohort of UK adolescent patients presenting with pubertal delay, in order to validate the use of genetic analysis of known causal genes to confirm the diagnosis of IHH or SLDP. We also evaluated the utility of genetic criteria to assist clinicians in confirming the diagnosis of IHH.

## Subjects and methods

### Patients

This study investigated a cohort of patients who were referred for genetic evaluation for delayed puberty from Paediatric Endocrinology and Paediatric services around the UK from 2015 to 2020 under a NIHR clinical research network portfolio study (Genetic Factors Affecting the Timing of Puberty, CPMS ID 30730).

Delayed puberty was defined as the onset of Tanner stage G2 (testicular volume > 3 mL) at >14 years in boys or Tanner stage B2 at > 13.0 year in girls (i.e. two s.d. later than average pubertal development). Chronic illness was excluded by detailed medical history, physical examination, and routine laboratory investigations, and hormonal investigations (basal and stimulated serum LH and FSH, serum testosterone, serum oestradiol, inhibin B) were evaluated to determine the clinical diagnosis. Pubertal progression was also assessed to establish the clinical diagnosis: those who were near to completion or had completed pubertal development (Tanner staging of at least G4 or B4) by 18 years of age and not requiring further sex steroid treatment were diagnosed as SLDP, whereas patients who had not completed puberty by the age of 18 years or had arrested puberty during prior to the age of 18 years were defined as IHH. Patients who did not have a definite diagnosis of SLDP or IHH, as they were still undergoing a period of clinical follow-up, were excluded from this analysis (*n* = 1).

### DNA sequencing and bioinformatics

This study utilised whole-exome sequencing (WES) data of 46 patients with central pubertal delay (i.e. SLDP or IHH), and WES data of 35 healthy control individuals with normal pubertal timing. WES was performed on DNA extracted from peripheral blood leukocytes, using an Agilent V5 platform and Illumina HiSeq 2000 sequencing. Fraction of target regions with coverage > 4× was 99.4–99.8%. The exome sequences were aligned to the UCSC hg19 reference genome using the Burrows–Wheeler Aligner software (BWA-MEM (bwa-0.7.12)). Picard tools (picard-tools-1.119) were used to sort alignments and mark PCR duplicates. The genome analysis toolkit (GATK-3.4-46) was used to realign around indels and recalibrate quality scores using dbSNP, Mills and 1000 genomes as reference resources. Variant calling and joint genotyping using pedigree information were performed using HaplotypeCaller in GVCF mode from the genome analysis toolkit. The resulting variants were filtered using the variant quality score recalibration (VQSR) function from GATK.

**Figure 1:**
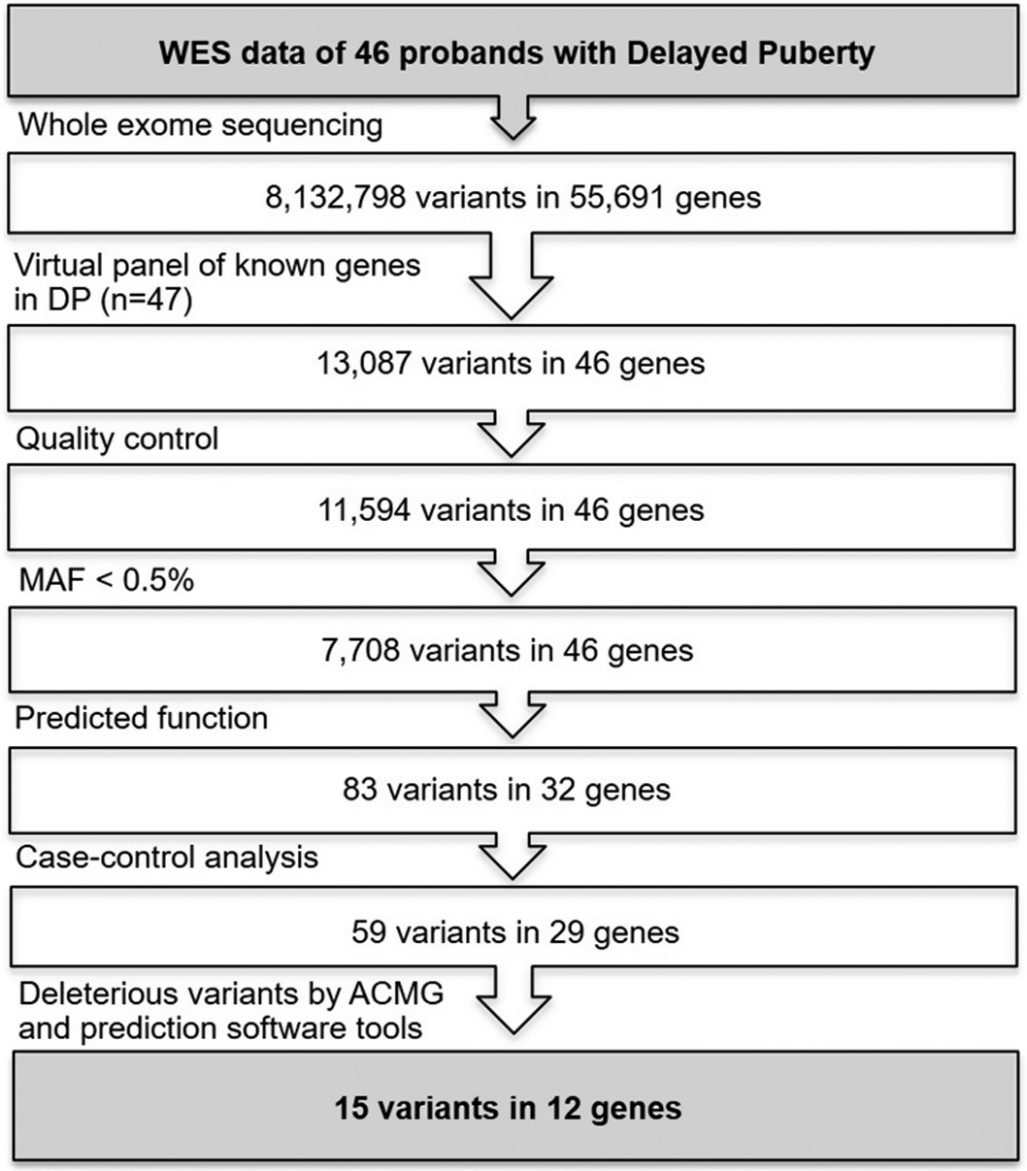
The analytic pipeline used for identifying genetic defects in patients with pubertal delay, using known genes reported in IHH or SLDP. MAF, minor allele frequency; DP, delayed puberty.

Analysis of the called variants was performed using Ingenuity Variant Analysis (QIAGEN Redwood City, www.qiagen.com/ingenuity). Filtering for potential causal variants was carried out using a classic bioinformatic pipeline (Fig. 1). A virtual panel of 47 genes, previously reported in the literature to have a causal role in the pathogenesis of delayed puberty (Table 1), including IHH (13, 18) and SLDP (9, 10, 14, 19, 20, 21), was applied as a filter across the whole-exome sequencing dataset. Quality control filters included thresholds for call quality, read depth and Phred strand bias, and only variants with minor allele frequency (MAF) < 0.5% in the Genome Aggregation Database (gnomAD) database (accessed March 2021) were retained. Predicted functional annotation prioritised nonsense, exonic missense, splice site variants, structural or promoter changes. Case-control analysis identified variants present in affected individuals and not present in controls, using a control group of 35 individuals previously whole-exome sequenced using the same bioinformatics pathway, who have been accurately phenotyped as having timing of puberty within the normal range. To identify likely disease-causing variants in these 47 genes, only variants that met the ACMG criteria (22) for pathogenicity, likely pathogenicity, or variants of uncertain significance (VUS) were selected in the analysis.

**Table 1:** List of the genes previously reported causing IHH and SLDP used as a virtual panel during WES analysis (9, 10, 12, 13, 14, 18, 19, 20, 21, 41).

**Phenotype**	*n*	Genes
IHH	36	*ANOS1, CCDC141, DCC/NTN1, DMXL2, FEZF1, FGF17, FGF8, FGFR1, FSHB, GNRH1, KISS1, KISS1R, KLB, LEP, LEPR, LHB, NSMF, NR0B1, NTN1, OTUD4, PCSK1, PLXNA1, PNPLA6, POLR3A/B, PROK2, PROKR2, RNF216, SEMA3E, SMCHD1, SOX10, SOX2, STUB1, TUBB3, WDR11*
IHH and SLDP	7	*HS6ST1, GNRHR, IL17RD, TAC3, TACR3, SEMA3A, CHD7*
SLDP	4	*EAP1, IGSF10, LGR4, FTO*

VUS identified were further selected for being potentially deleterious using *in silico* analysis software tools: SIFT (23), PolyPhen-2 (24), Mutation Taster (25), REVEL (26), and MetaLR (27). Variants predicted to be deleterious by ≥3 from five tools were retained (Fig. 1). Promoter variants were annotated using RegulomeDB (https://regulomedb.org/regulome-search) and Haploreg (https://pubs.broadinstitute.org/mammals/haploreg/haploreg.php) resources.

### Genotypic criteria for the diagnosis of IHH

Using the filtered WES data, the genotype of each patient was determined to support a genotypic diagnosis of either SLDP or IHH. This categorisation was done without consideration of clinical data to minimise the risk of bias in genotype interpretation. The genotype interpretation was assisted by bioinformatic tools and published literature. Information pertaining to previously reported mutations, including variant location in the protein structure and inheritance characteristics of variants in each gene, was reviewed. The pattern of inheritance for each variant in both the proband with pubertal delay and family relatives was used to determine whether the variant might cause IHH or SLDP (Supplementary Table 1, see section on supplementary materials given at the end of this article).

A genotypic diagnosis of IHH was made if the patient carried (i) a known deleterious variant in a known IHH gene with the same zygosity as previously demonstrated to cause IHH, (ii) a new predicted deleterious variant in a known IHH gene with the same zygosity as previously demonstrated to cause IHH, (iii) homozygosity for a new predicted deleterious variant where heterozygosity or homozygosity of this gene is reported in IHH.

A genotypic diagnosis of SLDP was made if the patient carried a known or new predicted deleterious variant in a known SLDP gene with the same zygosity as previously demonstrated to cause SLDP.

An inconclusive genotype was called if (i) no variants were found, or (ii) a new predicted deleterious variant in a known IHH gene with discordant zygosity to that previously demonstrated for example, heterozygous where homozygous carriage has been shown to cause IHH, or (iii) the oligogenic carriage of two separately predicted deleterious variants in two IHH genes or a mix of IHH and SLDP genes were identified.

### Ethical approval and consent to participate

Ethical approval was granted by the London–Chelsea NRES committee (13/LO/0257). All participants provided written informed consent prior to study participation. The study was conducted in accordance with the guidelines of The Declaration of Helsinki.

### Statistical analysis

Continuous data were expressed as mean and s.d. when normally distributed or median with interquartile range (IQR) otherwise. Fisher’s exact test was used to compare categorical variables in epidemiological data. Unpaired *t*-test (two-tail) or, for multiple comparisons, the Mann–Whitney *U-*test, was used to compare continuous variables as appropriate. Cohen’s kappa coefficient (*κ*) was used to demonstrate the correlation between genotypic and clinical diagnoses. Statistical differences were deemed significant at a *P*-value < 0.05. Statistical analysis was performed using GraphPad Prism 8 (GraphPad Software).

## Results

### Clinical characteristics of SLDP and IHH patient groups are similar at presentation

From 46 patients presenting at initial assessment with delayed puberty, 54.3% (*n = *25) had SLDP, and 45.7% (*n = *21) had IHH as their final clinical diagnosis. The majority were male accounting for 87% (*n = *40/46) and gender distributions were not different between SLDP and IHH groups. All clinical details are shown in Table 2. The median age at presentation was 16.0 (IQR 15.0, 17.1) years, with the majority of patients in early puberty, demonstrated by 40% of male patients with ≤3 mL and 47.5% with 4–9 mL testicular volumes, 34.8% of male and female patients with prepubertal pubic hair (PH1) Tanner stage and 50% with PH2, and 50% of female patients with prepubertal breast (B1) stage and 16.7% with B2 at first clinical assessment. Age at the first clinical sign of puberty (achievement of G2 or B2) was 15.6 vs 16.4 years in males and 11.8 vs 16.3 years in females for SLDP and IHH groups, respectively. A family history of pubertal delay was identified in 72% of those in the SLDP final clinical diagnostic group and 47.6% of the IHH group (*P-*value* = *0.2). Micropenis with bilateral cryptorchidism was found in two patients with a final clinical diagnosis of IHH, and of these two patients, one also demonstrated synkinesis, whilst a third patient had a cleft palate. Anosmia was found in five patients, all in the IHH group (0% vs 25%, P-value = 0.03); anosmia was, therefore, present in 25% of IHH patients. All patients had normal basal pituitary function (thyroid function tests, cortisol, IGF1, and prolactin). Thus, most patients in this cohort presented with isolated delayed puberty without clear clinical signs of IHH (e.g. micropenis, cryptorchidism or synkinesis), representative of the DP patients in whom the distinction between SLDP and IHH is considered most difficult.

**Table 2:** Demographic data and clinical details of pubertal delay patients both from the total cohort and compared between final clinical diagnosis of IHH and SLDP.

	Total (*n* =46)	Final clinical diagnosis		*P-*value
		SLDP (*n* =25)	IHH (*n* =21)	
Patients with identified variants, *n* (%)	15 (32.6)	6 (24.0)	9 (42.9)	0.2
Gender, *n* (%)				0.1
Male	40 (87.0)	24 (96.0)	16 (76.2)	
Female	6 (13.0)	1 (4.0)	5 (23.8)	
Ethnicity				0.5
European	29	16	13	
South Asian	6	2	4	
Ashkenazi Jewish	5	5	0	
Middle Eastern	2	2	0	
Turkish	2	0	2	
African	2	0	2	
Age at Tanner stage II (years)				
Male	15.9 (1.5)	15.6 (1.4)	16.4 (1.6)	0.1
Female	15.4 (2.3)	11.8 (0.0)	16.3 (1.2)	0.03*
Hormonal treatment, *n* (%)	40 (87.0)	21 (84.0)	19 (90.5)	0.8
Anosmia, *n* (%)	5 (10.9)	0 (0.0)	5 (23.8)	0.04*
MPH	173.4 (7.9)	173.5 (7.77)	173.3 (8.44)	0.9
MPH SDS	−0.41 (0.93)	−0.48 (1.06)	−0.30 (0.73)	0.6
Family history of DP, *n* (%)	28 (60.9)	18 (72.0)	10 (47.6)	0.2
Consanguinity, *n* (%)	1 (2.2)	0 (0.0)	1 (4.8)	0.9
IHH red flag signs, *n* (%)	2 (6.5)	0 (0.0)	3 (14.3)	0.9
First visit*				
Age (years)^ǂ^	16.0 (15.0, 17.1)	15.6 (14.9, 16.3)	16.5 (15.7, 17.2)	0.05
Weight SDS	−1.19 (1.78)	−1.58 (1.79)	−0.69 (1.69)	0.1
Height SDS	−1.67 (1.15)	−1.80 (1.10)	−1.51 (1.22)	0.4
BMI SDS	−0.29 (1.87)	−0.67 (1.79)	0.18 (1.90)	0.139
Tanner staging				
TV (mL), *n* (%)				0.2
≤ 3	16 (40.0)	7 (29.2)	9 (56.2)	
4–9	19 (47.5)	14 (58.3)	5 (31.2)	
10–15	5 (12.5)	3 (12.5)	2 (12.6)	
Genital staging, *n* (%)				0.1
I	16 (40.0)	11 (45.8)	5 (31.2)	
II	16 (40.0)	6 (25.0)	10 (62.5)	
III	5 (12.5)	5 (20.8)	0 (0.0)	
IV	2 (5.0)	1 (4.2)	1 (6.2)	
V	1 (2.5)	1 (4.2)	0 (0.0)	
Pubic hair staging, *n* (%)				0.3
I	16 (34.8)	9 (36.0)	7 (35.0)	
II	23 (50.0)	14 (56.0)	9 (45.0)	
III	3 (6.5)	0 (0.0)	3 (15.0)	
IV	1 (2.2)	1 (4.0)	0 (0.0)	
V	3 (6.5)	1 (4.0)	1 (5.0)	
Breast staging, *n* (%)				0.8
I	3 (60.0)	1 (100.0)	2 (50.0)	
II	1 (20.0)	0 (0.0)	1 (25.0)	
III	1 (20.0)	0 (0.0)	1 (25.0)	
S-LH (IU/L)^ǂ^	0.8 (0.2, 1.8)	0.8 (0.2, 2.1)	1.0 (0.1, 1.6)	0.6
S-FSH (IU/L)^ǂ^	1.8 (1.1, 2.8)	2.0 (1.6, 3.4)	1.5 (0.7, 2.7)	0.07
S-Test (nmol/L)^ǂ^	0.7 (0.4, 1.5)	1.0 (0.6, 4.9)	0.4 (0.4, 0.7)	0.007*
Peak S-LH (IU/L)^ǂ^	9.1 (1.7, 13.3)	11.70 (8.12, 14.25)	6.00 (1.17, 10.72)	0.09
Inhibin B (pg/mL)^ǂ^	51.0 (18.5, 115.0)	149.0 (104.0, 183.0)	36.0 (14.5, 69.8)	0.009*

*First visit data were missing from one female with IHH; ^ǂ^Data presented as median (IQR), otherwise presented as mean (s.d.).

DP, delayed puberty; MPH, mid parental height; SDS, standard deviation score; S-FSH, serum follicle-stimulating hormone; S-LH, serum luteinizing hormone; S-Test, serum testosterone; TV, testicular volume; BII/GII, Tanner stage II.

### Multiple deleterious variants in known pubertal delay genes are identified from whole-exome sequencing of patients with delayed puberty

**Figure 2:**
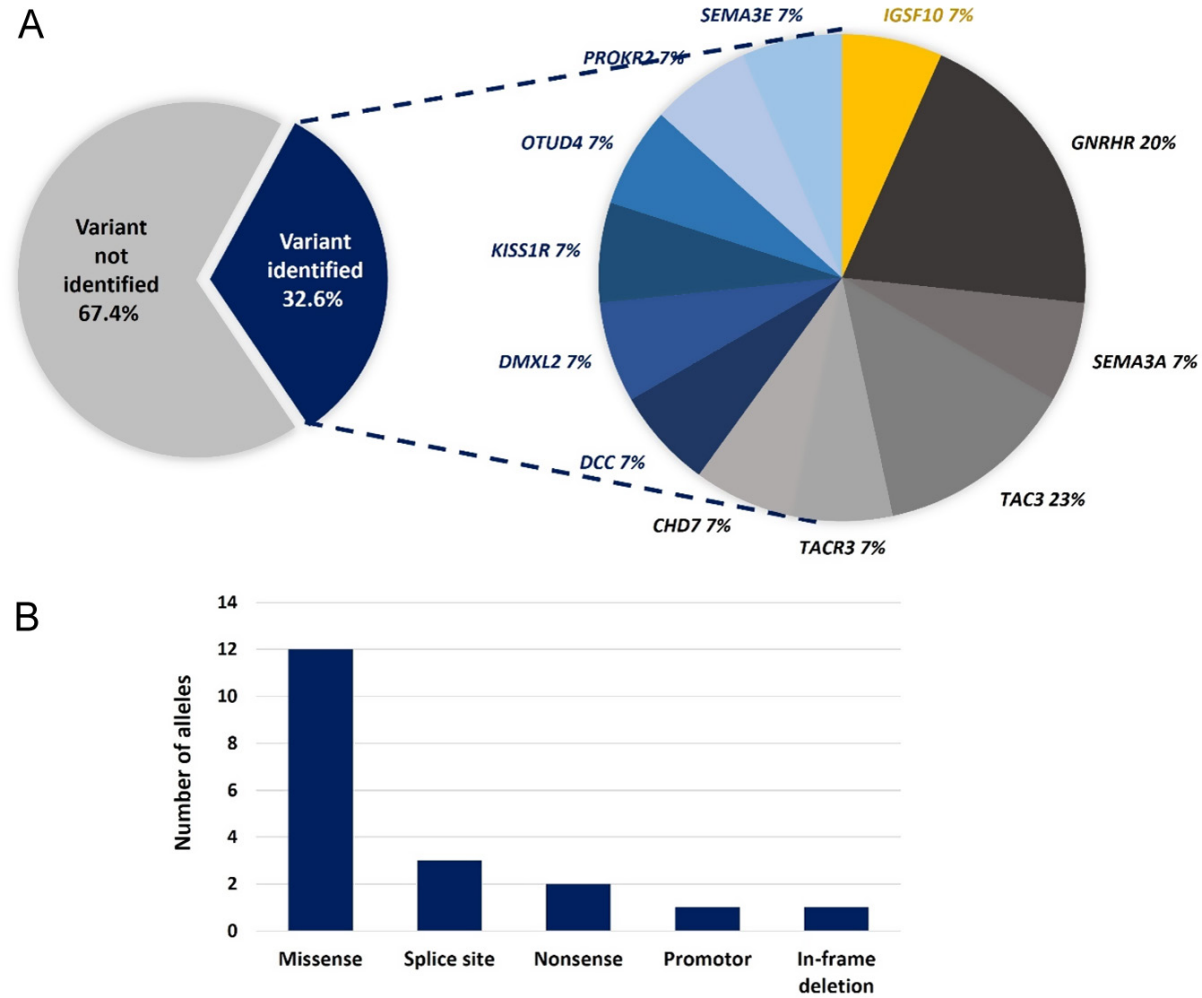
Variants identified in this cohort. (A) Proportion of variants identified in each gene group; yellow, genes confined to SLDP; blue, variants in genes reported in IHH only; grey, variants in genes reported in both SLDP and IHH. (B) Number of alleles in each variant category.

Of the 46 patients analysed by WES, 15 patients (32.6%) were identified with potentially deleterious variants in a known gene reported in either IHH or SLDP (Fig. 2). In all, 15 potentially deleterious variants in 12 genes were identified, with two siblings (patient 13 and 14) carrying the same variant, (*GNRHR* c.317A>G; p.Q106R). All variants were rare with a MAF of less than 0.5%, and the majority, 73.3% (*n = *11/15), were inherited in a heterozygous manner. The exception to this was variants in *GNRHR* (c.317A>G; p.Q106R), *PROKR2* (c.809G>A; p.R270H) and *TAC3* (c.209-1G>C), which displayed homozygous inheritance. One variant was identified in a gene confined to SLDP, *IGSF10* (1 of 4 genes in this category, 25%). Five of the genes reported in both SLDP and IHH were identified with eight variants in total (5 of 7 genes in this category, 71%), and six variants were identified in six genes previously reported only in IHH (9 of 35 genes in this category, 17.1%) (Fig. 2A). The most common type of allelic variation was missense (63.2%) followed by variants affecting a splice site (15.8%), nonsense (10.6%), promoter (5.3%) and in-frame deletion (5.3%) variants (Fig. 2B). The *KISS1R* promoter variant (c.-249G>A) occurred within a region with a highly significant puberty-specific differential promoter methylation pattern (28), predicted to be a site of EZH2 transcription factor binding, a member of the polycomb group of transcriptional regulators (29). The details of each deleterious variant, including zygosity, MAF and predicted functional impact are described in Table 3.

**Table 3:** Identified variants in pubertal delay patients.

Gene lists/genes	Variants	Protein variants	Reference	Translational impact	Zygosity	ACMG crtieria	SIFT	PolyPhen-2	Mutation Taster	REVEL	MetaLR
SLDP only											
*IGSF10*	c.7124A>G	p.N2375S		Missense	Het	VUS	X	X	X	X	X
Both SLDP and IHH											
*GNRHR*											
	c.317A>G	p.Q106R	(42, 43)	Missense	Hom	P	X	X	X	X	O
	c.436C>T	p.P146S	(44)	Missense	Het	LP	X	X	X	X	X
*SEMA3A*	c.1849C>T	p.R617Ter		Nonsense	Het	P	–	–	X	–	–
*TAC3*											
	c.209-1G>C	–	(31)	Splice site	Hom	P	–	–	X	–	–
	c.*2-1G>T	–		Splice site	Het	LP	–	–	–	–	–
*TACR3*	c.1090C>T	p.R364Ter		Nonsense	Het	P	–	–	X	–	–
*CHD7*	c.3738G>A	p.M1246I		Missense	Het	VUS	X	X	X	X	X
IHH only											
*DCC*	c.1933C>T	p.P645S	(45)	Missense	Het	VUS	X	X	X	O	O
*DMXL2*	c.2540C>T	p.T847I		Missense	Het	VUS	X	X	X	O	O
*KISS1R*	c.-249G>A	–		Promoter	Het	VUS	–	–	–	–	–
*OTUD4*	c.458_460delCTG	p.A153del		In-frame deletion	Het	VUS	–	–	–	–	–
*PROKR2*	c.809G>A	p.R270H	(46)	Missense	Hom	VUS	X	X	X	X	X
*SEMA3E*	c.398G>T	p.C133F		Missense	Het	VUS	X	X	X	X	X

X, a potentially deleterious variant by prediction tools (deleterious by SIFT, probably or possibly damaging by PolyPhen2, disease causing by Mutation Taster, likely disease causing by REVEL, and damaging by MetaLR); O, non-pathogenic predicted by prediction tools (tolerated by SIFT, benign or unknown by PolyPhen2, polymorphism by Mutation Taster, likely benign by REVEL, and tolerated by MetaLR); Het, heterozygous; Hom, homozygous; P, pathogenic; LP, likely pathogenic; VUS, variant of uncertain significance.

### Genotypic characteristics are distinct between IHH and SLDP patient groups

We compared the genetic characteristics between SLDP and IHH patients. In patients with IHH, an underlying genetic variant was identified more frequently (9/21, 42.9%) than in those with SLDP (6/25, 24.0%), but the difference was not significant (*P  = 0.2*). Deleterious variants previously reported only in SLDP were not identified in IHH patients. Homozygous, promoter and nonsense variants were identified only in patients with a final clinical diagnosis of IHH. Patients with a clinical diagnosis of SLDP were found to carry potentially deleterious variants in genes previously reported in IHH only, including *DMXL2, OTUD4*, and* SEMA3E* (Table 4).

**Table 4:** Comparison of genetic characteristics between patients clinically diagnosed as SLDP and IHH.

	Final clinical diagnosis	
	SLDP (%)	IHH (%)
Patient with variant identified		
Yes	6 (24.0)	9 (42.9)
No	19 (76.0)	12 (57.1)
Variants from gene list		
SLDP only	1 (14.2)	0 (0.0)
SLDP and IHH	2 (42.9)	6 (66.7)
IHH only	3 (42.9)	3 (33.3)
Zygosity of the variants		
Heterozygous	6 (100.0)	5 (55.6)
Homozygous	0 (0.0)	4 (44.4)
Variant category (*n* = 20 alleles)		
Predicted to affect protein structure/expression (nonsense, splice site, promoter)	1 (16.7)	5 (38.5)
Predicted not to affect protein structure/expression (missense, in-frame variant)	5 (83.3)	8 (61.5)

### Genetic criteria can be utilised for the diagnosis of IHH and SLDP in patients presenting with delayed puberty

After a list of qualified variants was filtered, each patient’s genotypic diagnosis was determined based on the criteria outlined in methods. One patient was found to have a genotypic diagnosis of SLDP, seven patients had a genotypic diagnosis of IHH, and the remaining seven had an inconclusive genotype (Table 5). Notably, three patients who were initially given a clinical diagnosis of SLDP (patients 10, 13, and 14) were shown to have a diagnosis of IHH by genetic analysis. At final diagnosis, these three patients had a confirmed clinical diagnosis of IHH after completion of follow-up. These three cases are in keeping with the published literature of these known pathogenic mutations, where cases with IHH can present initially with simple delayed puberty (30, 31). Parents of these cases, as in the literature, were either unaffected carriers or manifested self-limited delayed puberty, Supplementary Table 3 (30, 32). Autosomal recessive inheritance is a known inheritance pattern in IHH, but phenotypically, these cases are at the milder end of the spectrum, not associated classically with red flag signs for IHH, and thus are more likely to be misdiagnosed clinically. Homozygosity in the absence of consanguinity for these families is due, for the *GNRHR*_Q106R variant, to its MAF of 0.4% in the non-Finnish European population, and, for the *TAC3* splice variant to it being a founder mutation in the Congolese population, from where the patient’s parents both originated, Supplementary Table 3.

**Table 5:** The association between final clinical and genotypic diagnosis. Het, heterozygous; Hom, homozygous. Genotypic diagnosis is shown in bold, where discordant with the initial clinical diagnosis but concordant with final clinical diagnosis.

No.	Initial clinical diagnosis	Final clinical diagnosis	Sex	Age at 1st visit (years)	DP in family	Gene	Ethnicity	Nucleotide change	Protein change	MAF (%) by ethnicity	Zygosity	Genotypic diagnosis
1	Uncertain	SLDP	M	18.3	No	*IGSF10*	Non-Finnish European	c.7124A>G	p.N2375S	0.0256	Het	SLDP
2	Uncertain	SLDP	M	13.3	No	*DMXL2*	Askenazi Jewish	c.2540C>T	p.T847I	0	Het	Inconclusive
3	Uncertain	SLDP	M	17.2	No	*OTUD4*	Non-Finnish European	c.458_460delCTG	p.A153del	0.00558	Het	Inconclusive
4	Uncertain	SLDP	M	17.4	No	*CHD7*	Non-Finnish European	c.3738G>A	p.M1246I	0	Het	Inconclusive
5	Uncertain	SLDP	M	17.2	Father, uncle	*SEMA3E*	Non-Finnish European	c.398G>T	p.C133F	0	Het	Inconclusive
6	Uncertain	SLDP	M	15.0	Father, uncle	*TAC3*	African	c.*2-1G>T	Splice site	0.0259	Het	Inconclusive
7	Uncertain	IHH	M	15.6	Father	*PROKR2*	South Asian	c.809G>A	p.R270H	0.0588	Hom	IHH
8	Uncertain	IHH	M	14.8	Mother	*KISS1R*	Non-Finnish European	c.-249G>A	Promoter	0	Het	Inconclusive
9	Uncertain	IHH	M	15.9	Parents, brother	*SEMA3A*	Non-Finnish European	c.1849C>T	p.R617Ter	0.00088	Het	IHH
10	SLDP	IHH	F	14.9	Mother, sister	*TAC3*	African	c.209-1G>C	Splice site	0.112	Hom	**IHH**
11	Uncertain	IHH	M	18.1	Father	*GNRHR*	Non-Finnish European	c.436C>T	p.P146S	0.119	Het	Inconclusive
12	Uncertain	IHH	M	17.1	No	*DCC*	South Asian	c.1933C>T	p.P645S	0.173	Het	IHH
13	SLDP	IHH	M	16.5	Mother, brother	*GNRHR*	Non-Finnish European	c.317A>G	p.Q106R	0.418	Hom	**IHH**
14	SLDP	IHH	M	13.5	Mother, brother	*GNRHR*	Non-Finnish European	c.317A>G	p.Q106R	0.418	Hom	**IHH**
15	Uncertain	IHH	M	16	No	*TACR3*	South Asian	c.1090C>T	p.R364Ter	0.00327	Het	IHH

### A correlation between genotypic and clinical diagnosis was identified for patients presenting with delayed puberty

**Figure 3:**
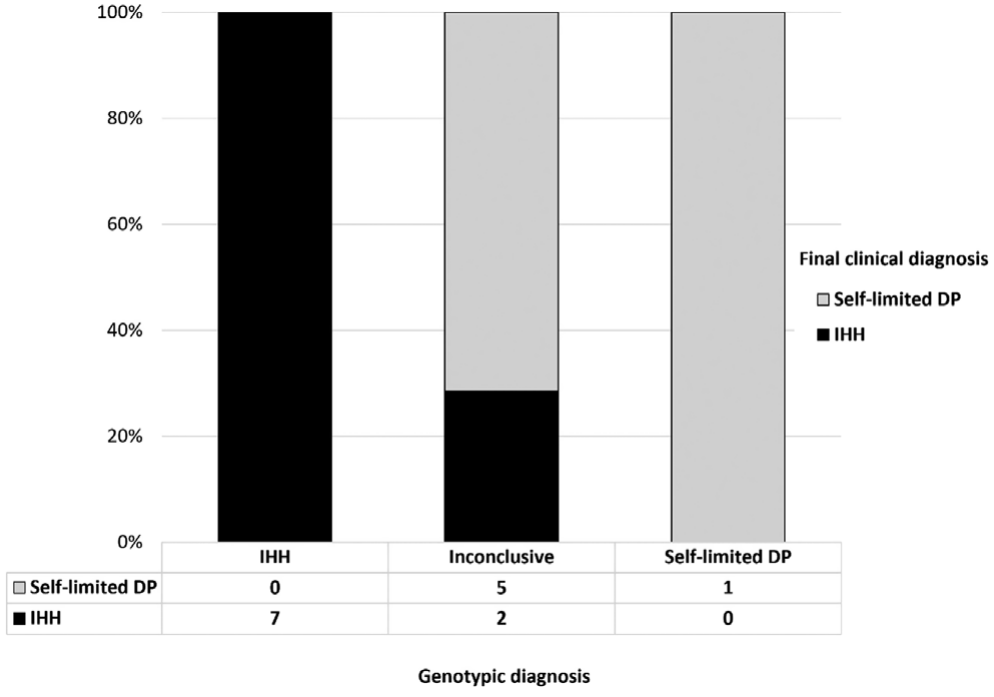
Clinical diagnosis of the patients who underwent WES with variants identified and grouped by genotypic criteria (*n = *19). X-axis shows three groups of genotypic diagnosis in patients who underwent WES. Y-axis shows the percentage of patients in each genotype diagnosis. Clinical diagnosis is shown by bars as indicated.

The correlation between final clinical diagnosis and genotype was assessed in 15 patients who were definitively diagnosed with either SLDP or IHH following the diagnostic criteria outlined above. Notably, the patients who were diagnosed with SLDP and IHH by genotyping had SLDP and IHH phenotypes, respectively, with a Cohen’s kappa (k) = 0.327 (95% IC, 0.137–0.517), demonstrating that genotype diagnosis has a fair agreement with the final clinical diagnosis (Fig. 3). Patients diagnosed as IHH by genotypic criteria are thus very likely to have IHH as a final clinical diagnosis. Likewise, patients with a genotype of SLDP are likely to have a clinical diagnosis of SLDP. For those patients who were diagnosed with an inconclusive genotype by WES, they may belong to the clinical phenotypic group of either SLDP or IHH.

Using this genotypic diagnostic framework, 7 of 21 patients with a final clinical diagnosis of IHH can be diagnosed with IHH, and all patients with a final clinical diagnosis of SLDP had a genetic result that was not compatible with IHH. Applying these criteria resulted in a sensitivity and specificity of 33.3% and 100%, respectively, of using genotypic criteria for diagnosis of IHH, with a positive predictive value (PPV) of 100%, and negative predictive value (NPV) of 64.10% (Supplementary Table 2).

## Discussion

Pubertal delay can be the presentation of a broad spectrum of clinical phenotypes ranging from IHH, which is a pathological condition and needs intensive medical therapy, to SLDP, a more benign condition usually compatible with normal reproductive capacity post-puberty. Many clinical and biochemical parameters have been applied to try to distinguish these two conditions; however, these all have limitations in terms of specificity and sensitivity (3). Diagnostic uncertainty is associated with increased psychological stress for both adolescents and their parents (33). In this study, we investigated for the first time whether the identification of a genetic defect in patients with pubertal delay, through WES combined with a virtual panel, can distinguish these two conditions.

Prompt diagnosis will aid clinical management, by ameliorating the need for patients to undergo unnecessary investigations or inappropriate treatment. Furthermore, early diagnosis of IHH can facilitate the use of optimal therapeutic modalities for pubertal induction, such as the use of gonadotropins in males with IHH and commencement of therapy at an earlier age than the standard sex steroid therapy indicated for SLDP patients (6). Our clinical data highlight this issue, as the mean age of development of secondary sexual characteristics, following hormonal induction, in the patients with a final diagnosis of IHH was over 16 years.

The findings of this study point to a fair correlation between genotypic diagnosis and final clinical diagnosis, with a 100% specificity and PPV of genetic testing for the diagnosis of patients with IHH. This study also found that patients who carry homozygous or loss-of-function variants in genes reported in IHH will be very likely to have a final clinical diagnosis of IHH. On the other hand, patients carrying variants reported only in SLDP with heterozygous inheritance are likely to have a clinical diagnosis of SLDP. From this cohort review, we found that testing through WES with a virtual panel can make a definitive diagnosis for 17.4% (*n = *8/46) of patients presenting with pubertal delay, and that of these patients, seven out of eight had IHH. This supports the use of genetic investigation in the clinical setting, combined with clinical and biochemical criteria, to confirm the diagnosis of IHH in adolescents presenting with pubertal delay.

Moreover, genetic testing also has a benefit for initial diagnosis in patients who do not manifest obvious clinical signs of IHH as we identified a genotypic diagnosis of IHH in three patients in whom there had been an initial clinical diagnosis of SLDP, who went on to have a final clinical diagnosis of IHH. In these patients, who presented with isolated DP without red flags for IHH, where there was also a family history of DP, a default diagnosis of SLDP was made. Such individuals may also have reasonable prepubertal testes volumes (e.g. 3 mL) or have entered puberty and then arrested, and without genetic analysis, it is only after several years of careful follow-up that they can be diagnosed as IHH.

This study also identified deleterious variants in three other genes known to contribute to IHH, namely* DMXL2, OTUD4* and *SEMA3E,* in three patients with a final clinical diagnosis of SLDP. Although these patients had clinical characteristics of SLDP, they may have a more significant defect in their GnRH neuroendocrine system, including impact on fertility or timing of menopause/andropause, which may need monitoring into adulthood. Moreover, it suggests that in a subset of patients with pubertal delay, there may be some overlap of genetic and pathophysiological mechanisms between SLDP and IHH, or lie along a spectrum of GnRH deficiency. This sub-category may be reflected by the ‘inconclusive genotypic’ group identified in this study, in whom a moderate burden of mutations in GnRH deficiency genes may lead to a SLDP phenotypic pattern, whilst a severe mutational burden, such as homozygous or loss-of-function mutation, may lead to a more severe reproductive phenotype, that is, IHH. It is likely that with further genetic discovery and a better understanding of the pathophysiology underlying these conditions, the genotype–phenotype correlation in this inconclusive group will become clearer. We believe that this group merits careful observation of their clinical progression.

Our study has some limitations. The number of genes in the virtual panel associated with SLDP (*n = *4) was far smaller than those associated with IHH (*n = *42). Only a few causal genes have been identified in SLDP to date, leading to a lower pick-up rate for SLDP mutations in this study (deleterious variants were identified in 25% (1 in 4) SLDP genes and 33% (14 in 43) of IHH genes in this study). Therefore, our group is working to better characterise the genetic basis of SLDP. Identification of a larger number of SLDP genes and their use in the virtual panel will improve the ability to make a genetic diagnosis in SLDP patients at presentation. Moreover, genotypic interpretation in patients with oligogenic inheritance is complex because of our lack of knowledge of variant–variant interaction. We have described such patients’ genotypes as inconclusive to minimise potential bias. Furthermore, although some patients had a family history of DP, DNA from the majority of these family members was not available to enable an analysis of genotype–phenotype correlation in the wider pedigree. Finally, this genetic analysis shows lower sensitivity and negative predictive value than some biochemical modalities (3, 34, 35, 36, 37, 38). Given that a combination of investigations can increase the sensitivity and specificity to diagnosis IHH (39, 40), this type of genetic analysis is at present likely to be best combined with biochemical profiling (e.g. basal LH, FSH, inhibin B, AMH) in order to maximise the diagnostic accuracy.

To our knowledge, this is the first study to demonstrate the correlation between genotypic diagnosis and final clinical diagnosis in a cohort of adolescent patients with severe pubertal delay, validating the use of genetic analysis to support the distinction between the clinical diagnosis of SLDP and IHH. We have also described a set of genotypic criteria for interpreting WES results from a virtual panel using curated information from previous reports. The use of early genetic diagnosis in this condition has the potential for significant cost savings as it can prevent unnecessary investigations and lead to improved health and fertility outcomes for patients. In summary, our analysis shows that WES analysis using a virtual panel in patients with delayed puberty is a useful tool to give a definite diagnosis in an uncertain clinical presentation.

## Supplementary Material

Table S1 Genotype diagnosis by variant using literature review of each gene and variant that have been previously reported in IHH/KS, self-limited DP and unaffected individuals. Het: heterozygous, Hom: homozygous, KS: Kallmann syndrome, ID: Intellectual disability, SLDP: self-limited DP, SNHL: sensorineural hearing loss

Table S2. 2X2 table for analysis of the utility of genotypic diagnosis to diagnosis IHH in patients with pubertal delay (n=46)

Table S3. Clinical data for family members of probands 10, 13 and 14

## Declaration of interest

The authors declare that there is no conflict of interest that could be perceived as prejudicing the impartiality of this study.

## Funding

T S is funded by Faculty of Medicine, Prince of Songkla University. M T D receives funding from the Great Ormond Street Hospital (GOSH) Children’s Charity and the Medical Research Foundation, UK (grant# 535963). Research at GOSH benefits from funding received from the NIHR Biomedical Research Centre (MTD). S R H is funded by the National Institute for Health Research [CL-2017-19-002] and the Rosetrees Trust [M222-F1].
