# Supplementary material for: Genetic evaluation supports differential diagnosis in adolescent patients with delayed puberty
Source: Eur J Endocrinol. 2021 Aug 17;185(5):617–27. doi: 10.1530/EJE-21-0387 (PMC8558847; doi:10.1530/EJE-21-0387)
Supplement: Table S1 Genotype diagnosis by variant using literature review of each gene and variant that have been previously reported in IHH/KS, self-limited DP and unaffected individuals. Het: heterozygous, Hom: homozygous, KS: Kallmann syndrome, ID: Intellectual disability, SLDP: self-limited DP, SNHL: senso [file supplementary_table_1.pdf]

Table S1 Genotype diagnosis by variant using literature review of each gene and variant that have been previously reported in IHH/KS, self-limited DP and unaffected individuals. Het: heterozygous, Hom: homozygous, KS: Kallmann syndrome, ID: Intellectual disability, SLDP: self-limited DP, SNHL: sensorineural hearing loss

Genes	Associated phenotype	IHH	SLDP	Normal	Domain disease causing	Variants	Protein variants	Zygosity	Variant lies in specific-domain	Variant specific for phenotype	Genotype Dx
CHD7 (1-4)	CHARGE features	Het, oligogenic het	Het	Het	Mutations were found along the protein in both specific and non-specific domains	c.3738G>A	p.M1246I	Het	Non-specific	No	Inconclusive
DCC (5, 6)	Facial asymmetry, MR, obesity, SNHL	Het, oligogenic het	No	Het	5 mutations have been reported which are located in specific domains including Ig-like C2 domain type 2 and fibronectin type III domain 1, 3, 5	c.1933C>T	p.P645S	Het	Fibronectin type III domain 3	p.P645S (het) has KS	IHH

DMXL2 (7, 8)	none-autoimmune DM, demyelinating polyneuropathy, MR	Hom	No	Het	3 cases with homozygous deletion of 5 amino acid in exon 24 (c.5824_5838del, p.1942_1946del), which is in non-specific domain, have IHH<br>- Parents and a sister who carry heterozygous of the above mutation have normal pubertal timing	c.2540C>T	p.T847I	Het	non- specific domain	No	Inconclusive
GNRHR (9-14)	-	Hom, compound het	Het	Het	R262Q (hom) has been reported in CDGP	c.317A>G	p.Q106R	Hom	Extracellular loop domain2	-Patients with compound het of p.Q106R and other mutation in GnRH gene have been found to cause IHH<br>- Parent of proband who carries p.Q106R (het) has normal puberty	IHH
						c.436C>T	p.P146S	Het	Intracellular loop domain2	- 2 sisters carry p.P146S (het) have IHH<br>- Mother of 2 sisters carries same mutation	Inconclusive
IGSF10 (15)	-	No	Het, digenic het	Het	2 het mutations in leucine-rich repeats (LRR) domains 1-7 have been reported to cause self-limited DP	c.7124A>G	p.N2375S	Het	Ig domain	No	SLDP

KISS1R (16-19)	-	Het, hom, compound het	Het	Het	More than 30 mutations have been reported to cause DP. The zygosity includes heterozygous, homozygous or compound heterozygous mutation.<br>- Some variants in heterozygous state such as p.A287E, p.P476R do not cause IHH.	c.-249G>A	-	Het	96 base pairs before transcription start	No	Inconclusive
OTUD4 (20, 21)	Progressive dementia & ataxia	Digenic hom	No	Het	- Digenic hom in RNF216 and OTUD4 cause IHH<br>- Individuals with het mutation are unaffected	c.458_460delCTG	p.A153del	Het	Within catalytic domain	No	Inconclusive
PROKR2 (22-27)	-	Hom, digenic het & het	No	no	Most mutations cause IHH/KS	c.809G>A	p.R270H	Hom	3rd intracellular loop domain	-This variant (het) with *SEMA3A* p.R617Q (het) has been found with IHH phenotype	IHH
SEMA3A (28-30)	-	Het, oligogenic het	No	No	Heterozygous mutation of *SEMA3A* located along the gene have been found to cause IHH/KS.	c.1849C>T	p.R617Ter	Het	Ig domain	p.R617Q mutation combined with heterozygous mutation of *CCDC141* and *PROKR2* was found in IHH.	IHH
SEMA3E (31, 32)	-	Oligogenic het	No	No	Oligogenic het mutation in SEMA and Ig domain have been found in IHH/KS.	c.398G>T	p.C133F	Het	SEMA domain	Mutation in the same domain, p.M102T, has been found in KS when combine with heterozygous mutations of	Inconclusive

										PLNXA1 and *CCDC141*.	
TAC3 (33-38)	-	Hom, digenic het	Het	No	Patient with IHH has homozygous or digenic heterozygous mutation of this gene.<br>- This gene is also found in family members of probands with IHH. The reported mutations are missense and mutation in splice site of TAC3.	c.209-1G>C		Hom	-	Heterozygous splice site mutation (TAC3 g.18595G>T) has been reported in individuals with delayed puberty; homozygous carriage of this variant has been reported in individuals with IHH with reversal	IHH
						c.*2-1G>T		Het	-		SLDP
TACR3 (33, 35-38)	-	Hom, compound het, het	Het	Het	- Mutation in this gene have been found in both IHH and self-limited DP. Heterozygous mutation of this gene can cause both conditions.	c.1090C>T	p.R364Ter	Het	Intracellular region	No	IHH

References

1. Bartels CF, Scacheri C, White L, Scacheri PC, Bale S. Mutations in the CHD7 gene: the experience of a commercial laboratory. Genet Test Mol Biomarkers. 2010;14(6):881-91.

2. Bergman JE, Janssen N, van der Sloot AM, de Walle HE, Schoots J, Rendtorff ND, Tranebjaerg L, Hoefsloot LH, van Ravenswaaij-Arts CM, Hofstra RM. A novel classification system to predict the pathogenic effects of CHD7 missense variants in CHARGE syndrome. Human mutation. 2012;33(8):1251-60.

3. Gonçalves CI, Patriarca FM, Aragüés JM, Carvalho D, Fonseca F, Martins S, Marques O, Pereira BD, Martinez-de-Oliveira J, Lemos MC. High frequency of CHD7 mutations in congenital hypogonadotropic hypogonadism. Sci Rep. 2019;9(1):1597.

4. Wu J, Zhao Y, Wang X, Jiang F, Hou Q, Chen DN, Zheng R, Yu R, Zhou W, Li JD, et al. Phenotypic spectrum of idiopathic hypogonadotropic hypogonadism patients with CHD7 variants from a large Chinese cohort. The Journal of clinical endocrinology and metabolism. 2019.

5. Manitt C, Mimee A, Eng C, Pokinko M, Stroh T, Cooper HM, Kolb B, Flores C. The netrin receptor DCC is required in the pubertal organization of mesocortical dopamine circuitry. The Journal of neuroscience : the official journal of the Society for Neuroscience. 2011;31(23):8381-94.

6. Bouilly J, Messina A, Papadakis G, Cassatella D, Xu C, Acierno JS, Tata B, Sykiotis G, Santini S, Sidis Y, et al. DCC/NTN1 complex mutations in patients with congenital hypogonadotropic hypogonadism impair GnRH neuron development. Human molecular genetics. 2018;27(2):359-72.

7. Sedwick C. A Vesicular Protein Important for Puberty. PLOS Biology. 2014;12(9):e1001953.

8. Tata B, Huijbregts L, Jacquier S, Csaba Z, Genin E, Meyer V, Leka S, Dupont J, Charles P, Chevenne D, et al. Haploinsufficiency of Dmxl2, Encoding a Synaptic Protein, Causes Infertility Associated with a Loss of GnRH Neurons in Mouse. PLOS Biology. 2014;12(9):e1001952.

9. Hietamäki J, Hero M, Holopainen E, Känsäkoski J, Vaaralahti K, Iivonen AP, Miettinen PJ, Raivio T. GnRH receptor gene mutations in adolescents and young adults presenting with signs of partial gonadotropin deficiency. PloS one. 2017;12(11):e0188750.

10. Ulloa-Aguirre A, Janovick J, Leaños-Miranda A, Conn P. Misrouted cell surface GnRH receptors as a disease aetiology for congenital isolated hypogonadotropic hypogonadism. Human reproduction update. 2004;10:177-92.

11. Vagenakis GA, Sgourou A, Papachatzopoulou A, Kourounis G, Papavassiliou AG, Georgopoulos NA. The gonadotropin-releasing hormone (GnRH)-1 gene, the GnRH receptor gene, and their promoters in patients with idiopathic hypogonadotropic hypogonadism with or without resistance to GnRH action. Fertility and sterility. 2005;84(6):1762-5.

12. Vaaralahti K, Wehkalampi K, Tommiska J, Laitinen EM, Dunkel L, Raivio T. The role of gene defects underlying isolated hypogonadotropic hypogonadism in patients with constitutional delay of growth and puberty. Fertility and sterility. 2011;95(8):2756-8.

13. Fathi AK, Hu S, Fu X, Huang S, Liang Y, Ning Q, Luo X. Molecular defects of the GnRH-receptor gene in Chinese patients with idiopathic hypogonadotropic hypogonadism and the severity of hypogonadism. Journal of pediatric endocrinology & metabolism : JPEM. 2012;25(7-8):659-68.

14. Choi J-H, Balasubramanian R, Lee PH, Shaw ND, Hall JE, Plummer L, Buck CL, Kottler M-L, Jarzabek K, Wołczynski S, et al. Expanding the Spectrum of Founder Mutations Causing Isolated Gonadotropin-Releasing Hormone Deficiency. The Journal of Clinical Endocrinology & Metabolism. 2015;100(10):E1378-E85.

15. Howard SR, Guasti L, Ruiz-Babot G, Mancini A, David A, Storr HL, Metherell LA, Sternberg MJ, Cabrera CP, Warren HR, et al. IGSF10 mutations dysregulate gonadotropin-releasing hormone neuronal migration resulting in delayed puberty. 2016;8(6):626-42.

16. Alzahrani AJ, Ahmad A, Alhazmi T, Ahmad L. An Isolated Hypogonadotropic Hypogonadism due to a L102P Inactivating Mutation of KISS1R/GPR54 in a Large Family. Case Rep Pediatr. 2019;2019:3814525.

17. Chelaghma N, Rajkanna J, Trotman J, Fuller G, Elsey T, Park SM, Oyibo SO. Normosmic idiopathic hypogonadotrophic hypogonadism due to a rare KISS1R gene mutation. Endocrinol Diabetes Metab Case Rep. 2018;2018.

18. Francou B, Paul C, Amazit L, Cartes A, Bouvattier C, Albarel F, Maiter D, Chanson P, Trabado S, Brailly-Tabard S, et al. Prevalence of KISS1 Receptor mutations in a series of 603 patients with normosmic congenital hypogonadotrophic hypogonadism and characterization of novel mutations: a single-centre study. Human reproduction (Oxford, England). 2016;31(6):1363-74.

19. Nalbantoğlu Ö, Arslan G, Köprülü Ö, Hazan F, Gürsoy S, Özkan B. Three Siblings with Idiopathic Hypogonadotropic Hypogonadism in a Nonconsanguineous Family: A Novel KISS1R/GPR54 Loss-of-Function Mutation. Journal of clinical research in pediatric endocrinology. 2019;11(4):444-8.

20. Margolin DH, Kousi M, Chan YM, Lim ET, Schmahmann JD, Hadjivassiliou M, Hall JE, Adam I, Dwyer A, Plummer L, et al. Ataxia, dementia, and hypogonadotropism caused by disordered ubiquitination. The New England journal of medicine. 2013;368(21):1992-2003.

21. Zhao Y, Mudge MC, Soll JM, Rodrigues RB, Byrum AK, Schwarzkopf EA, Bradstreet TR, Gygi SP, Edelson BT, Mosammaparast N. OTUD4 Is a Phospho-Activated K63 Deubiquitinase that Regulates MyD88-Dependent Signaling. Mol Cell. 2018;69(3):505-16.e5.

22. Cox KH, Oliveira LMB, Plummer L, Corbin B, Gardella T, Balasubramanian R, Crowley WF. Modeling mutant/wild-type interactions to ascertain pathogenicity of PROKR2 missense variants in patients with isolated GnRH deficiency. Human molecular genetics. 2018;27(2):338-50.

23. Kim JH, Seo GH, Kim GH, Huh J, Hwang IT, Jang JH, Yoo HW, Choi JH. Targeted Gene Panel Sequencing for Molecular Diagnosis of Kallmann Syndrome and Normosmic Idiopathic Hypogonadotropic Hypogonadism. Experimental and clinical endocrinology & diabetes : official journal, German Society of Endocrinology [and] German Diabetes Association. 2019;127(8):538-44.

24. Senthilraja M, Chapla A, Jebasingh FK, Naik D, Paul TV, Thomas N. Parallel Multi-Gene Panel Testing for Diagnosis of Idiopathic Hypogonadotropic Hypogonadism/Kallmann Syndrome. Case Rep Genet. 2019;2019:4218514.

25. Zhao Y, Wu J, Jia H, Wang X, Zheng R, Jiang F, Chen DN, Chen Z, Li JD. PROKR2 mutations in idiopathic hypogonadotropic hypogonadism: selective disruption of the binding to a Gα-protein leads to biased signaling. FASEB journal : official publication of the Federation of American Societies for Experimental Biology. 2019;33(3):4538-46.

26. Zhou C, Niu Y, Xu H, Li Z, Wang T, Yang W, Wang S, Wang DW, Liu J. Mutation profiles and clinical characteristics of Chinese males with isolated hypogonadotropic hypogonadism. Fertility and sterility. 2018;110(3):486-95.e5.

27. Dodé C, Teixeira L, Levilliers J, Fouveaut C, Bouchard P, Kottler ML, Lespinasse J, Lienhardt-Roussie A, Mathieu M, Moerman A, et al. Kallmann syndrome: mutations in the genes encoding prokineticin-2 and prokineticin receptor-2. PLoS genetics. 2006;2(10):e175.

28. Hanchate NK, Giacobini P, Lhuillier P, Parkash J, Espy C, Fouveaut C, Leroy C, Baron S, Campagne C, Vanacker C, et al. SEMA3A, a gene involved in axonal pathfinding, is mutated in patients with Kallmann syndrome. PLoS genetics. 2012;8(8):e1002896.

29. Young J, Metay C, Bouligand J, Tou B, Francou B, Maione L, Tosca L, Sarfati J, Brioude F, Esteva B, et al. SEMA3A deletion in a family with Kallmann syndrome validates the role of semaphorin 3A in human puberty and olfactory system development. Human reproduction (Oxford, England). 2012;27(5):1460-5.

30. Dai W, Li JD, Zhao Y, Wu J, Jiang F, Chen DN, Zheng R, Men M. Functional analysis of SEMA3A variants identified in Chinese patients with isolated hypogonadotropic hypogonadism. Clinical genetics. 2020.

31. Kotan LD, Isik E, Turan I, Mengen E, Akkus G, Tastan M, Gurbuz F, Yuksel B, Topaloglu AK. Prevalence and associated phenotypes of PLXNA1 variants in normosmic and anosmic idiopathic hypogonadotropic hypogonadism. Clinical genetics. 2019;95(2):320-4.

32. Cariboni A, André V, Chauvet S, Cassatella D, Davidson K, Caramello A, Fantin A, Bouloux P, Mann F, Ruhrberg C. Dysfunctional SEMA3E signaling underlies gonadotropin-releasing hormone neuron deficiency in Kallmann syndrome. The Journal of clinical investigation. 2015;125(6):2413-28.

33. Topaloglu AK, Reimann F, Guclu M, Yalin AS, Kotan LD, Porter KM, Serin A, Mungan NO, Cook JR, Imamoglu S, et al. TAC3 and TACR3 mutations in familial hypogonadotropic hypogonadism reveal a key role for Neurokinin B in the central control of reproduction. Nat Genet. 2009;41(3):354-8.

34. Zhu J, Choa RE, Guo MH, Plummer L, Buck C, Palmert MR, Hirschhorn JN, Seminara SB, Chan YM. A shared genetic basis for self-limited delayed puberty and idiopathic hypogonadotropic hypogonadism. The Journal of clinical endocrinology and metabolism. 2015;100(4):E646-54.

35. Francou B, Bouligand J, Voican A, Amazit L, Trabado S, Fagart J, Meduri G, Brailly-Tabard S, Chanson P, Lecomte P, et al. Normosmic congenital hypogonadotropic hypogonadism due to TAC3/TACR3 mutations: characterization of neuroendocrine phenotypes and novel mutations. PloS one. 2011;6(10):e25614.

36. Root AW. Reversible isolated hypogonadotropic hypogonadism due to mutations in the neurokinin B regulation of gonadotropin-releasing hormone release. The Journal of clinical endocrinology and metabolism. 2010;95(6):2625-9.

37. Tusset C, Noel SD, Trarbach EB, Silveira LFG, Jorge AAL, Brito VN, Cukier P, Seminara SB, Mendonça BBd, Kaiser UB, et al. Mutational analysis of TAC3 and TACR3 genes in patients with idiopathic central pubertal disorders. Arquivos brasileiros de endocrinologia e metabologia. 2012;56(9):646-52.

38. Gianetti E, Tusset C, Noel S, Au M, Dwyer A, Hughes V, Abreu AP, Carroll J, Trarbach E, Silveira L, et al. TAC3/TACR3 Mutations Reveal Preferential Activation of Gonadotropin-Releasing Hormone Release by Neurokinin B in Neonatal Life Followed by Reversal in Adulthood. The Journal of clinical endocrinology and metabolism. 2010;95:2857-67.
