# Supplementary material for: Genetic evaluation supports differential diagnosis in adolescent patients with delayed puberty
Source: Eur J Endocrinol. 2021 Aug 17;185(5):617–27. doi: 10.1530/EJE-21-0387 (PMC8558847; doi:10.1530/EJE-21-0387)
Supplement: Table S2. 2X2 table for analysis of the utility of genotypic diagnosis to diagnosis IHH in patients with pubertal delay (n=46) [file supplementary_table_2.pdf]

Table S2. 2X2 table for analysis of the utility of genotypic diagnosis to diagnosis IHH in patients with pubertal delay (n=46)

		**Final clinical diagnosis**	
		**IHH (n=21)**	**SLDP (n=25)**
**Genotypic diagnosis**	**IHH**	7	0
	**Non-IHH**	14	25
