# Supplementary material for: Genetic evaluation supports differential diagnosis in adolescent patients with delayed puberty
Source: Eur J Endocrinol. 2021 Aug 17;185(5):617–27. doi: 10.1530/EJE-21-0387 (PMC8558847; doi:10.1530/EJE-21-0387)
Supplement: Table S3. Clinical data for family members of probands 10, 13 and 14 [file supplementary_table_3.pdf]

Table S3. Clinical data for family members of probands 10, 13 and 14

Proband 10						
Proband Genotype	Minor allele frequency	Maternal phenotype	Maternal genotype	Paternal phenotype	Paternal genotype	Consanguinity
TAC3_ c.209-1G>C Homozygous	0.1% African/ African American; known founder mutation in the Congolese population (1)	Delayed puberty, AAM 15.5 yrs	TAC3_ c.209-1G>C Heterozygous	Not available	Not available	Not known, but parents both from families originally from the same geographical region of Congo
Proband 13 & 14						
Genotype	Minor allele frequency	Maternal phenotype	Maternal genotype	Paternal phenotype	Paternal genotype	Consanguinity
GNRHR_ c.317A>G_p.Q106R Homozygous	0.4% Non-European Finnish	Delayed puberty, B2 14 yrs, AAM 16 yrs	GNRHR_ c.317A>G_ p.Q106R Heterozygous	Self-recalled normal age of puberty onset	GNRHR_ c.317A>G_ p.Q106R Heterozygous	Nil

Reference

1. Young J, Bouligand J, Francou B, Raffin-Sanson ML, Gaillez S, Jeanpierre M, et al. TAC3 and TACR3 defects cause hypothalamic congenital hypogonadotropic hypogonadism in humans. The Journal of clinical endocrinology and metabolism. 2010;95(5):2287-95.
